# Otolaryngology Training in Uganda: The Mbarara University of Science and Technology Experience

**DOI:** 10.1002/oto2.30

**Published:** 2023-03-02

**Authors:** Ryan A. Bartholomew, John Ceremsak, Victoria Nyaiteera, Eva Senechal, Vivek Kanumuri, Mack Cheney, Ronald K. de Venecia, Doreen Nakku, David A. Shaye

**Affiliations:** ^1^ Department of Otolaryngology, Massachusetts Eye and Ear Harvard Medical School Boston Massachusetts USA; ^2^ Department of Otolaryngology Mbarara University of Science and Technology Mbarara Uganda

**Keywords:** global surgery, low‐middle income country, otolaryngology, residency training, surgical training, Uganda

## Abstract

Sub‐Saharan Africa has a high otolaryngologic disease burden exacerbated by an inadequate number of otolaryngologists. The Otolaryngology department at Mbarara University of Science & Technology in Uganda is addressing this problem by having created Uganda's second national residency training program in 2010. We chronicled an early period in the program's development by reporting surgical case quantity and complexity, as defined by “key indicator procedure” classification per the United States Accreditation Council for Graduate Medical Education, and interpreting it with respect to a timeline of significant events. Procedure complexity, but not total number per year, increased over the study period—KIPs increased from 3% in 2012 (6 of 175 total procedures) to 29% in 2016 (35 of 135 total procedures). During this period of complexity increase, operating room capacity expanded, faculty received advanced training and increased in number, and operative equipment improved.

The considerable otolaryngologic disease burden^1^, ^2^, ^3^ in Sub‐Saharan Africa coupled with inadequate numbers of otolaryngologists has led to an “otolaryngology workforce crisis.”^4^ In 2015, Uganda had 35 otolaryngologists and 2 otolaryngology training centers for approximately 35 million people.^5^, ^6^ Strengthening local training programs can increase the number of otolaryngologists.^7^ Mbarara University of Science and Technology (MUST) is a public university in southwestern Uganda with an affiliated teaching regional referral hospital,^8^ and in 2010 created the country's second otolaryngology residency program.

In the United States, otolaryngology residents must complete a minimum number of “Key Indicator Procedures” (KIPs), defined by the Accreditation Council for Graduate Medical Education (ACGME), to graduate (Table 1).^9^ KIPs are a proxy for surgical complexity, although use in the assessment of Ugandan residents warrants caution given differences between the 2 countries. To chronicle the development of the MUST otolaryngology training program, we report surgical case quantity and complexity, as measured by KIPs, during an early 5‐year period and interpret it with respect to significant events.

**Table 1:** Summary of the Accreditation Council for Graduate Medical Education (ACGME) Key Indicator Procedure Guidelines for Otolaryngology (www.ACGME.org).^9^

Key indicator category	Procedure	Number required
Head & Neck Surgery	Parotidectomy	15
	Neck Dissection	27
	Oral Cavity	10
	Thyroid & Parathyroidectomy	22
Otology & Audiology	Tympanoplasty	17
	Mastoidectomy	15
	Ossicular Chain Surgery	10
Facial Plastic & Reconstructive Surgery	Rhinoplasty	8
	Craniomaxillofacial Trauma	12
	Flaps and Grafts	20
General & Pediatrics	Airway—Pediatric and Adult	20
	Congenital Masses	7
	Sinus (Ethmoidectomy)	40
	Bronchoscopy	22

## Materials and Methods

This study was approved by the MUST Ethical Review Board and received exempt status from the Massachusetts Eye and Ear (MEE) internal review board. Otolaryngologic procedures at MUST between 2012 and 2016 were characterized from surgical logbooks and coded per ACGME KIP criteria. Data for only the first 7 months of the 2016 year was available and used to extrapolate the remainder of the years data by assuming constant procedure rate. A narrative history was constructed through structured faculty interviews.

## Results

Inspired by the British system, Ugandan healthcare is free at the point of need with access determined by resource availability. MUST was founded in 1989. In the mid‐1990s, MUST began employing Cuban otolaryngologists on 2‐ to 3‐year contracts. In 2010 the residency program was established and at that time the department consisted of a single medical assistant, nurse, resident, and Cuban otolaryngologist who oversaw a twice weekly outpatient clinic. With only a single operating room in the hospital, otolaryngology surgical time was restricted to a half day per week and primarily reserved for urgent cases.

Otolaryngology residency training at MUST is 3 years with a competence‐based curricula and formalized didactics. The MUST program initially accepted 1 to 2 new residents per year but increased to 4 to 5 residents per year in 2016. Trainees are required to have completed a Bachelor of Medicine and Surgery (MB.ChB), or equivalent degree, and a clinical internship year. Residents defend a research dissertation in their final year. All residents have come from Uganda or nearby African countries and have established practices in their countries of origin.

**Figure 1:**
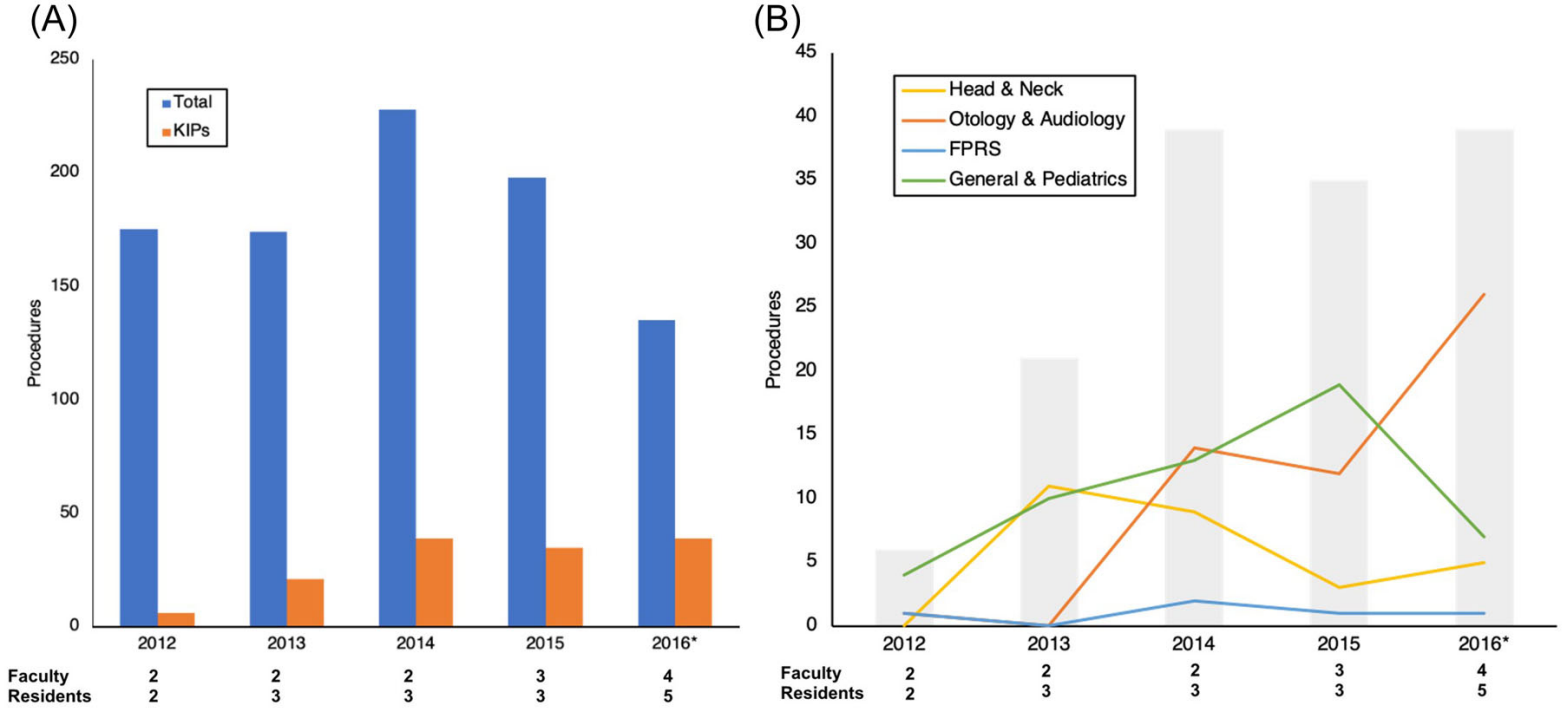
(A) Total procedures and key indicator procedures (KIPs) performed. (B) KIPs by category. Gray bars indicate total KIPs. *2016 data estimated from the first 7 months. Total faculty and resident numbers per year are shown.

During 2012 to 2016, the number of surgeries ranged from 135 to 228 per year (Figure 1A). Simpler surgeries, such as excisions of cutaneous lesions, nasal polypectomies, and adenotonsillectomies, initially predominated. KIPs increased from 6 in 2012 to 39 in 2016 (Figure 1B). While total procedures in 2016 was less than that in 2012, the percent of these which qualified as KIPs increased from 3% to 29% (Figure 1).

**Figure 2:**
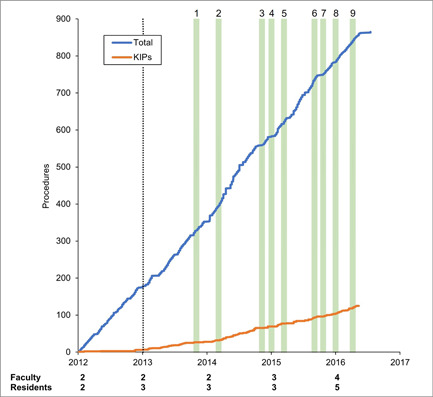
Cumulative procedures and key indicator procedures (KIPs) performed. Total number of faculty and residents per year are shown. Dotted line indicates increase in operating room availability. Green bars indicate interventions identified numerically in Table 2.

The rate of total procedures performed remained largely unchanged over the study period while the rate of KIPs performed increased (as indicated by the slope of the plotted lines in Figure 2). Notable developments included an increase in faculty (from 2 to 4) and of active trainees (from 2 to 5). A cadaveric surgical skills laboratory was built in 2015. Finally, beginning in 2013, dedicated time for otolaryngology procedures doubled from a half day to a full day per week concurrent with an increase in hospital capacity to 4 operating rooms and acquisition of a surgical microscope.

From 2013 to 2016, 9 educational interventions involving external otolaryngology programs occurred (Table 2). MUST faculty traveled to Kenya in 2013, 2015, and 2016 to participate in Head & Neck Surgery workshops. Faculty also completed 2 fellowships in Canada in 2013 and 2015 focusing on Otology and Rhinology, respectively. The faculty is now entirely comprised of African physicians, with the final Cuban otolaryngologist having left in 2018.

**Table 2:** Summary of Educational Interventions From Visiting Faculty During the Study Period

	Date	Institution	Clinical focus	Description
1.	Nov. 2013	MEEI	Laryngology	OR cases, didactics
2.	Mar. 2014	UBC	Rhinology, Otology & Audiology	OR cases, didactics, audiology practical, endoscopic nasal examination
3.	Nov. 2014	MEEI		
			FPRS	Facial nerve assessment, cadaveric dissection & flaps
4.	Jan. 2015	MEEI	Rhinology, Allergy	OR cases, didactics
5.	Mar. 2015	MEEI	Radiology	Didactics
6.	Sept. 2015	MEEI	Head & Neck	Facial nerve reanimation didactics, cadaveric dissections
7.	Oct. 2015	UBC & UM	Rhinology, Otology & Audiology	OR cases
8.	Jan. 2016	UBC	Head & Neck	OR cases, cadaveric dissections
9.	Apr. 2016	MEEI	Head & Neck	OR cases

Abbreviations: FPRS, facial plastic & reconstructive surgery; MEEI, Massachusetts Eye and Ear Infirmary; OR, operating room; UBC, University of British Columbia; UM, University of Manitoba.

## Conclusions

We assessed the growth of an otolaryngology program in Uganda over a 5‐year period using ACGME KIPs as a proxy for case complexity. During this time, MUST otolaryngologists performed hundreds of surgeries to the benefit of the regional community. While the number of procedures performed per year was approximately constant, procedure complexity increased substantially. This increase in complexity was concurrent with an increase in program resources—personnel, equipment, operating room access, and training.

Despite this, a resident training at MUST would not complete the minimum number of ACGME KIPs required in the United States. This may reflect the limited operating room resources, insufficient training, and differing prevalence of pathologies in Uganda. Also contributing are differences in pathologies treated, as oral cavity cancer and bony facial trauma are referred to the Uganda Cancer Institute and Mulago National Hospital in Kampala, respectively. Notably, the total number of procedures decreased from 2014 to 2016. This decrease, in combination with the increase in KIPs over the same period, may reflect MUST deferring simple cases to smaller neighboring private hospitals to prioritize more complex cases.

This study has limitations. First, ACGME KIPs may not reflect optimal KIPs for Ugandan trainees. Additionally, we cannot attribute causation between described events and surgeries performed. Future studies should explore the contributions of educational interventions and clinical resources towards surgeries performed by developing otolaryngology programs as well as formally define minimum competency criteria for these programs in low‐ and middle‐income countries.

## Author Contributions

**Ryan A. Bartholomew**, design, conduct, analysis, manuscript preparation; **John Ceremsak**, design, conduct, analysis, manuscript preparation; **Victoria Nyaiteera**, design, analysis, manuscript preparation; **Eva Senechal**, conduct, manuscript preparation; **Vivek Kanumuri**, design, manuscript preparation; **Mack Cheney**, design, manuscript preparation; **Ronald K. de Venecia**, design, manuscript preparation; **Doreen Nakku**, design, analysis, manuscript preparation; **David A. Shaye**, design, analysis, manuscript preparation.

## Disclosures

**Competing interests**: None.

## Funding source

None.
